# Coexistence of Superconductivity and Magnetic Ordering in the In–Ag Alloy Under Nanoconfinement

**DOI:** 10.3390/nano14221792

**Published:** 2024-11-07

**Authors:** Marina V. Likholetova, Elena V. Charnaya, Evgenii V. Shevchenko, Yurii A. Kumzerov, Aleksandr V. Fokin

**Affiliations:** 1 Physics Department, St. Petersburg State University, 198504 St. Petersburg, Russia; m.likholetova@spbu.ru (M.V.L.); e.shevchenko@spbu.ru (E.V.S.); 2 Ioffe Institute, 194021 St. Petersburg, Russia; yu.kumzerov@mail.ioffe.ru (Y.A.K.); midbarzin@yandex.ru (A.V.F.)

**Keywords:** nanostructured In–Ag alloy, superconductivity, magnetization, proximity effects, ferromagnetism induced at interface

## Abstract

The impact of the interface phenomena on the properties of nanostructured materials is the focus of modern physics. We studied the magnetic properties of the nanostructured In–Ag alloy confined within a porous glass. The alloy composition was close to the eutectic point in the indium-rich range of the phase diagram. Temperature dependences of DC magnetization evidenced two superconducting transitions at 4.05 and 3.38 K. The magnetization isotherms demonstrated the superposition of two hysteresis loops with low and high critical fields below the second transition, a single hysteresis between the transitions and ferromagnetism with weak remanence in the normal state of the alloy. The shape of the loop seen below the second transition, which closes at a low magnetic field, corresponded to the intermediate state of the type-I superconductor. It was ascribed to strongly linked indium segregates. The loop observed below the first transition is referred to as type-II superconductivity. The secondary and tertiary magnetization branches measured at decreasing and increasing fields were shifted relative to each other, revealing the proximity of superconducting and ferromagnetic phases at the nanometer scale. This phenomenon was observed for the first time in the alloy, whose components were not magnetic in bulk. The sign of the shift shows the dominant role of the stray fields of ferromagnetic regions. Ferromagnetism was suggested to emerge at the interface between the In and AgIn_2_ segregates.

## 1. Introduction

Nanostructured superconductors are the focus of modern physical research as they are very promising materials for various technical applications in microelectronics, robotics, and data processing [1,2]. Great attention is paid to superconducting nanostructures obtained by embedding metals and metallic alloys into nanoporous templates, in particular, into silica matrices (synthetic opals and porous glasses). The morphology of such nanostructures is driven by the geometry of the pore network and by the degree of the pore filling. Recent studies showed that nanoconfinement affects remarkably the properties of metals and alloys (see, for instance, [3,4,5,6,7,8,9]), in particular the superconducting properties [10,11,12,13,14]. In most cases, the porous template/metal nanostructures demonstrated features of dirty type-II superconductors with the upper critical fields, which are higher for smaller pore sizes [15,16]. The inversion of the type of superconductivity for single-component metals occurred due to the restriction of the free electron path under nanoconfinement. However, type-I superconductivity was also reported for synthetic opals loaded with tin [17]. Inherent inhomogeneities of nanostructures lead to strong pinning of superconducting vortices. Positive curvatures of the critical lines were found in some ranges of the *H*-*T* phase diagrams and were treated on the basis of the proximity effect. Magnetic instabilities were observed on the isotherms of magnetization for some nanostructures depending on particular metals and templates. New experimental opportunities were expected for more complicated nanostructures consisting of porous templates filled with metallic and ferromagnetic substances. In fact, it was shown in [18] that the extended metal/ferromagnetic interface within a porous glass loaded with indium and nickel caused a shift in the magnetization-versus-field hysteresis loops due to local magnetic fields. A great variety of superconducting phenomena found in nanostructures with metals and metallic alloys suggests that even more encouraging progress can be achieved in further studies.

Here, we present the results of magnetic measurements for a porous glass filled with the In–Ag alloy. Bulk In–Ag alloy, In–Ag films, and layered structures are considered for applications as low-temperature joints for superconductors, packaging, stacked-chip processes, and wafer-to-wafer bonding [19,20,21]. Previously, superconductivity was studied for the vapor-quenched In–Ag films and multilayers [22,23]. In our experiments, we monitored the temperature dependences of the DC magnetization at various magnetic fields for the porous glass/In–Ag alloy nanocomposite. Two superconducting transitions were observed. The DC magnetization isotherms were found to be a superposition of loops with smaller and higher critical magnetic fields. Anomalous shifts in the magnetization loops were revealed and were treated as a result of the stray fields caused by the emergence of the ferromagnetic order in the alloy under nanoconfinement.

## 2. Materials and Methods

**Figure 1:**
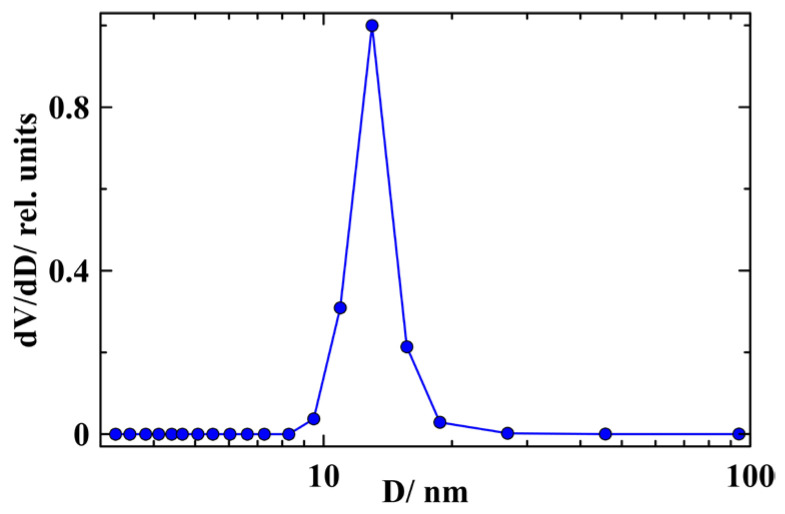
Pore size distribution in the porous glass.

The porous glass, which was used as a template, was obtained from a sodium borosilicate glass subjected to thermal treatment and acid leaching. The pore size and total pore volume were found by nitrogen porosimetry using a Quadrasorb SI analyzer (Quantachrome Instruments, Boynton Beach, FL, USA). The mean pore size was 13 nm. The pore size diagram is presented in Figure 1. The pore volume was equal to 21% of the porous glass bar. The melted In–Ag alloy was embedded into pores under high pressure of up to 20 kbar. The composition of the In–Ag was near the eutectic point with 5 at.% Ag. According to the alloy phase diagram [24,25,26], the solidus line in the In-rich composition range is at 417 K. Below the solidus, the In–Ag alloy consists of the intermetallic AgIn_2_ and In segregates. The pore filling with the In–Ag alloy was about 90%, as was found by weighing the empty and loaded glass bar. The thin plate for magnetic measurements was cut from the loaded porous glass. Its surface was cleaned thoroughly from the bulk alloy. The sample weight was equal to 7.37 mg.

The temperature dependences of the DC magnetization *M* were monitored using a MPSM SQUID-VSM (Quantum Design, San Diego, CA, USA) within a range of 1.8 to 10 K at magnetic fields *H* up to 70 kOe. The measurements were carried out upon warming after preliminary cooling in zero fields (ZFC protocol), upon successive cooling in fields (FCC protocol), and upon warming in fields (FCW protocol). The *M*(*H*) isotherms were measured at several temperatures, ramping the field in the range from −70 to 70 kOe. The plate was oriented in parallel to the magnetic field.

The X-ray powder diffraction pattern was obtained at room temperature using a D8 Discover (Bruker, Billerica, MA, USA) CuK_α_ diffractometer.

## 3. Results

**Figure 2:**
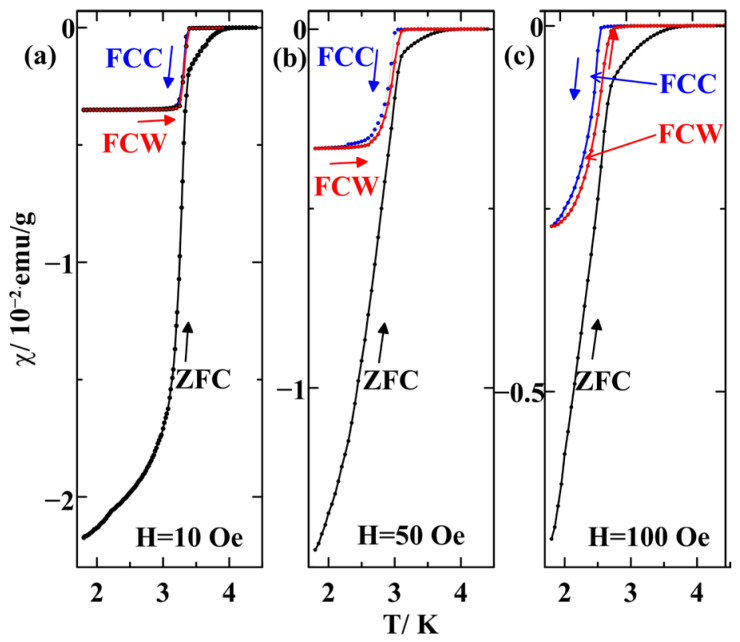
Temperature dependences of susceptibilities measured under the ZFC (black symbols and lines), FCC (blue symbols and lines), and FCW (red symbols and lines) protocols at fields of 10 (**a**), 50 (**b**), and 100 (**c**) Oe.

Temperature dependences of the susceptibility χ=M/H measured at the magnetic fields 10, 50, and 100 Oe are shown in Figure 2. One can see the onset of superconductivity at $T_{c1}$ = 4.05 K in 10 Oe. The ZFC magnetization decreases continuously below this temperature. The abrupt increase in the ZFC magnetization occurs at $T_{c2}$ = 3.38 K in 10 Oe. The behavior of the magnetization curves below $T_{c1}$ and below $T_{c2}$ is different. The screening of the applied field within the range between $T_{c1}$ and $T_{c2}$ is weak. Its maximum is lower by more than one order of magnitude than the maximal screening achieved near 1.8 K. The pronounced difference between the ZFC and FCC curves, as well as between the ZFC and FCW curves, within this temperature range evidences the type-II superconductivity and strong pinning of the superconducting vortices. The diamagnetic screening close to 1.8 K is near complete. Taking into account the pore filling factor and the pore volume, we estimated the value $4\pi \chi_{V}\approx$ −0.87 in a field of 10 Oe. Here, $\chi_{V}$ is the volume susceptibility. This shows that the superconducting currents enclose practically the whole sample volume. The difference between the susceptibilities obtained under the ZFC and FCC protocols below $T_{c2}$ is not so great, demonstrating weaker pinning. Moreover, the onset of superconductivity shifts slow to low temperatures with increasing the applied magnetic field, while the temperature of the pronounced change in the slope of the ZFC, FCC, and FCW curves decreases much faster. This signifies quite different critical fields. The distinct behavior of the magnetization curves below $T_{c1}$ and $T_{c2}$ allows us to suggest that we observe two superconducting transitions. The first transition occurs in a small fraction of the confined alloy, and the second transition occurs in the major volume of the alloy. In addition, it will be shown below that $T_{c2}$ corresponds to the emergence of type-I superconductivity, while $T_{c1}$ corresponds to the onset of type-II superconductivity. The first transition with the onset of superconductivity at $T_{c1}$ is quite diffuse. Figure 2 demonstrates also a thermal hysteresis between the FCC and FCW curves below the second transition, the FCW curve being closer to the ZFC one than the FCC curve.

**Figure 3:**
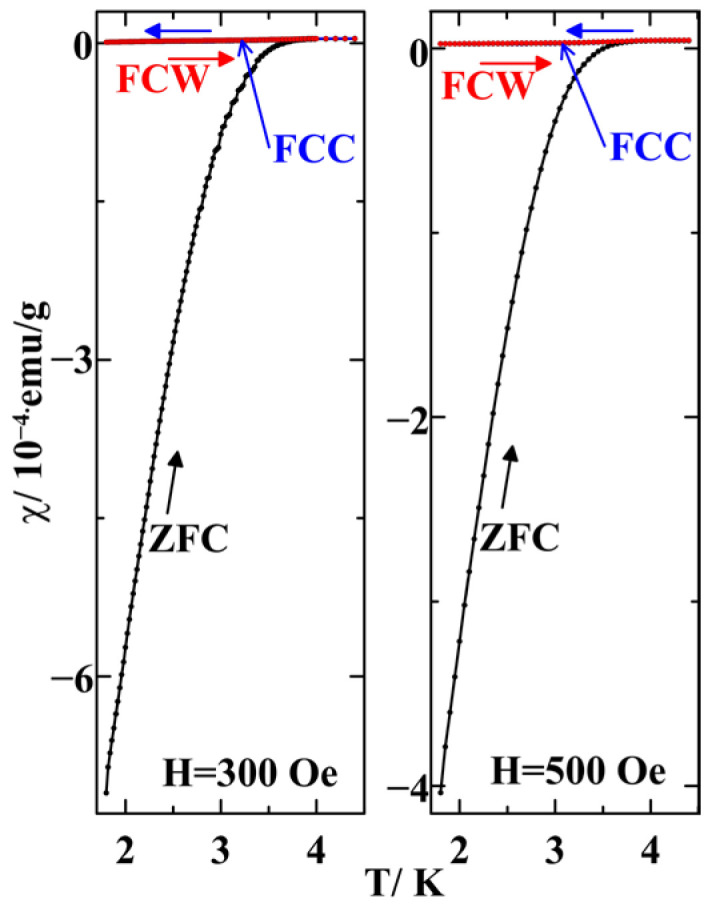
Temperature dependences of susceptibilities measured at 300 and 500 Oe under the ZFC (black symbols and lines), FCC (blue symbols and lines), and FCW (red symbols and lines) protocols.

Figure 3 presents temperature dependences of the susceptibility observed at the fields 300 and 500 Oe. We can see that the second superconducting transition moved below the low temperature limit of our equipment.

**Figure 4:**
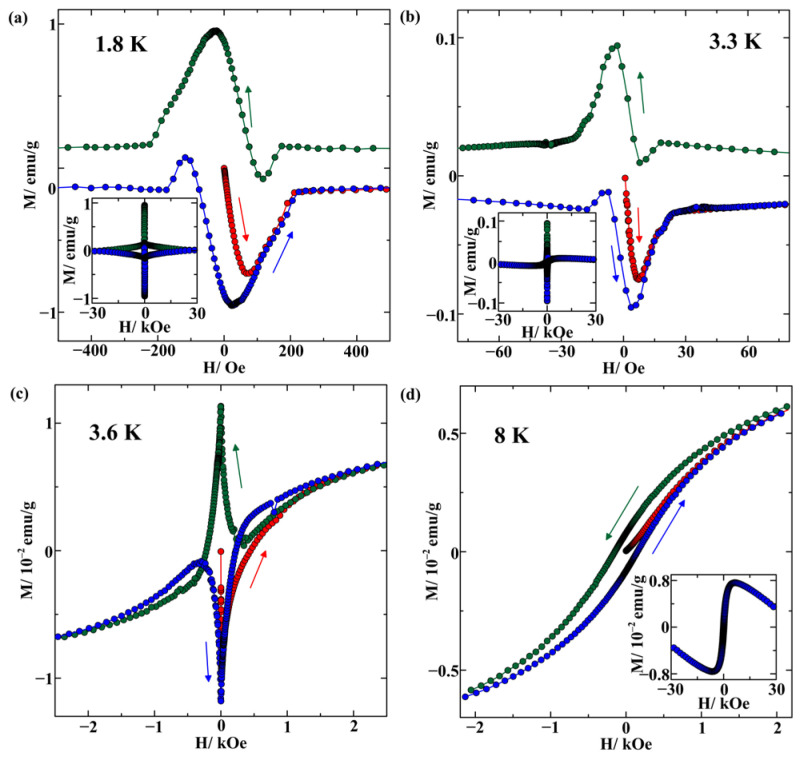
Central parts of isotherms of magnetizations obtained at temperatures 1.8 (**a**), 3.3 (**b**), 3.6 (**c**), and 8 (**d**) K. The arrows indicate the directions of ramping the field. The red, green, and blue symbols and lines correspond to the virgin, secondary, and tertiary magnetizations, respectively. The insets present the magnetization curves on a larger scale.

The *M*(*H*) isotherms obtained at 1.8, 3.3, 3.6, and 8 K are shown in Figure 4. The isotherms are corrected taking into account the residual fields in the superconducting magnet, which were evaluated using the Pd reference sample. The isotherms at 1.8 and 3.3 K (Figure 4a,b) look like the superposition of two hysteresis loops with higher and lower critical fields. The hysteresis loop at 3.6 K is simple. The corresponding critical field is much higher than the critical field for the central hysteresis loop at 1.8 K. Note the shifts in the secondary and tertiary magnetizations relative to each other in Figure 4a–c. For comparison, the initial and corrected magnetization isotherms for bulk In–Ag alloy at 1.8 K are presented in Figure S1 (in Supplementary Materials), which shows the absence of the shift between the secondary and tertiary curves after correcting the field.

The *M*(*H*) isotherm at 8 K, namely, above the first superconducting transition, evidences the existence of ferromagnetism in the sample under study. The emergence of ferromagnetism is confirmed by the temperature dependence of magnetization measured within the range from 1.8 to 300 K in a field of 500 Oe, which demonstrates the bifurcation of the ZFC and FCC curves (Figure S2 in Supplementary Materials). As can be seen in Figure 4d and Figure S2, the ferromagnetic order coexists with diamagnetic and paramagnetic contributions.

**Figure 5:**
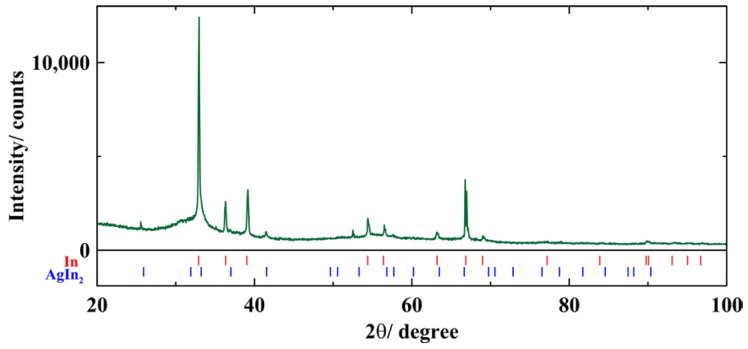
X-ray pattern of the porous glass/In–Ag alloy nanocomposite.

The X-ray powder pattern obtained at room temperature is shown in Figure 5. The pattern consists of peaks associated with peaks for bulk In with a space group *I*4/mmm (139) and AgIn_2_ intermetallic with a tetragonal space group *I*4/mcm (140). The base line also shows broad humps caused by the amorphous glass template.

## 4. Discussion

The bulk In–Ag alloy has a very complicated phase diagram [24,25,26]. In the indium-rich range of the alloy composition, the alloy solidifies with the formation of crystalline In and AgIn_2_ segregates. Figure 5 confirms that the In–Ag alloy within pores also consists of the same segregates as the bulk one. The comparatively narrow X-ray diffraction peaks in Figure 5 evidence that the segregate sizes are significantly greater than the pore size. The estimates of the segregate size from the peak broadening give values greater than 90 nm. The superconducting temperature for bulk indium is 3.414 K. The second transition has a very close temperature. Therefore, we can assume that the second superconducting transition is due to crystalline indium. The strong diamagnetic screening seen in Figure 2 below the second transition fully corresponds to high indium composition.

The origin of superconductivity below $T_{c1}$ is not as obvious. The weak diamagnetic screening below the first transition points out the small amount of the superconducting phase. However, superconductivity was not found in the AgIn_2_ intermetallic. Moreover, the first-principles electronic structure calculations predicted that the AgIn_2_ intermetallic can transform to the superconducting state at temperatures not higher than 3 K [27]. On the other hand, superconductivity below about 4.7 K was found in a vapor-quenched thin film of indium doped with silver [22] and was treated as a result of indium amorphization provoked by 12% silver addition. Note that the amorphous solid state, which was obtained for thick quench-condensed pure indium, demonstrated the onset of superconductivity near 4 K [28]. Thus, it can be that some tiny amount of amorphous indium is formed upon freezing the In–Ag alloy within pores and responds to weak superconductivity below 4.05 K. This suggestion agrees with findings for the critical field of the amorphous indium [28]. The critical field was estimated to be near 23 kOe at zero temperature. In our measurements shown in Figure 4, the critical field can be estimated as 23 kOe at 1.8 K. Another possibility to explain the appearance of the first superconducting transition in our sample can be related to the formation within pores of a small amount of fine indium that segregates very weakly linked with other indium. Actually, studies of superconductivity for indium under porous glass nanoconfinement showed a pronounced increase in the superconducting temperature up to 4 K [29]. Enhancement of the transition temperature was also found in earlier studies of indium embedded into a porous glass [16,30]. A rise in the superconducting temperature up to 4.1 K was observed for indium within opal pores [31]. It was emphasized in [31] that the increase in the superconducting transition temperature correlated to the increase in the critical field. Some increase in the critical temperature (however, much weaker) was also observed for isolated indium nanoparticles [32]. Note that the critical field found for indium nanoparticles within a porous glass was also much stronger than in bulk indium [29]. As we must expect the broad distribution of sizes of fine indium particles within pores, then the critical temperatures for them can vary following the size variations. This could explain the diffuse character of the first transition. The broad distribution of the critical temperatures should also be expected for the amorphous indium regions.

**Figure 6:**
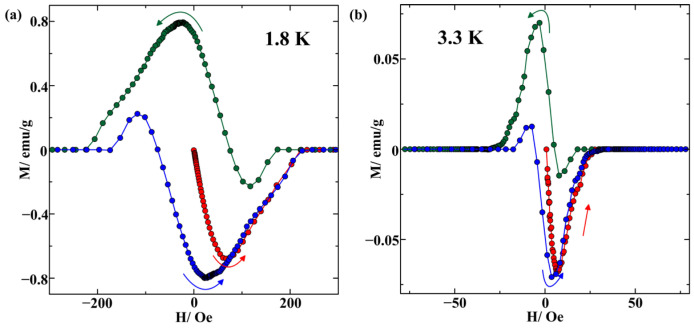
The separated central hysteresis loops for temperatures 1.8 (**a**) and 3.3 (**b**) K. The arrows indicate the directions of ramping the field. The red, green, and blue symbols and lines correspond to the virgin, secondary, and tertiary magnetizations, respectively.

The intricate shape of the *M*(*H*) isotherms presented in Figure 4 below $T_{c2}$ can be treated as a result of the superposition of two hysteresis loops. The central part of the hysteresis plot corresponds to crystalline indium segregates with the superconducting temperature $T_{c2}$. This central loop is not seen at 3.6 K, namely above $T_{c2}$. To see the central loop better, we separated this loop from the total isotherm at 1.8 and 3.3 K. The separated loops are shown in Figure 6. The loops are partly reversible in agreement with weak pinning observed in Figure 2. The critical field is low. It can be estimated from the virgin magnetization curves as 200 and 25 Oe at 1.8 and 3.3 K, respectively. These values practically coincide with the relevant critical magnetic fields found for bulk indium [33]. The coincidence of the critical fields for the central loop with those in bulk indium and the loop shape (according to the detailed analysis in [34]) along with the close similarity of $T_{c2}$ to the critical temperature in bulk indium and the weak difference between the ZFC and FC curves, indicate that indium segregates within the porous glass under study remain in the type-I superconducting state. This contrasts with the results obtained in [29], where the much stronger critical field implied the type-II superconductivity for a porous glass/In nanocomposite. This difference is most likely caused by better pore filling in the sample under study. Due to better connectivity of the indium network within pores, the mean electron-free path is not affected noticeably by nanoconfinement, and the coherence length is not shortened.

The most striking finding of the present study consists of the shift in the secondary and tertiary magnetization curves with respect to each other (Figure 4 and Figure 6). Such phenomena were not observed for bulk or nanostructured metals or metallic alloys apart from a porous glass sample loaded with metallic indium and ferromagnetic nickel [18]. While no theoretical models were developed to treat such nontrivial shapes of the hysteresis loops, we could suggest that the shift in the magnetization curves results from the coexistence of superconductivity and ferromagnetism on the nanoscale. The physics of the shift is assumed to be the same as the shifts in the critical current maxima found in heterostructures with superconducting and ferromagnetic layers [35,36,37]. While superconductivity and ferromagnetism are antagonists, they can coexist on the nanometer scale [35,38,39]. At present, unique properties of the superconductor-ferromagnetic heterostructures are the focus of modern studies due to their great importance for fundamental physics and applications. The shifts in the *M*(*H*) branches obtained upon increasing and decreasing magnetic field could be treated similarly to those of the Fraunhofer oscillations as a result of the proximity impact. The shift sign can be different depending on the dominant proximity effect and the geometry of heterostructures [40]. When the stray fields of ferromagnetics dominate, the secondary magnetization curve should be shifted to negative fields owing to positive remanence in ferromagnetics after saturation at strong positive external fields, while the tertiary magnetization curve shifts to positive fields. The short-range inverse proximity effect [41,42,43,44] and the electromagnetic proximity effect [45,46] lead to the opposite trend. Both signs of the shifts were shown in [35] for the Fraunhofer oscillations depending on the heterostructure morphology. Figure 6 shows that the magnetization branches of the central hysteresis loops below $T_{c2}$ shift to negative fields with respect to the virgin magnetization upon sweeping the field from positive to negative values. This evidences the dominance of the remanent magnetization in the ferromagnetic regions. The shift decreases weakly with increasing temperature. The hysteresis loop at 3.6 K (Figure 4c) is also affected by proximity to the ferromagnetic phase. In the range of low magnetic fields, the shift between the peaks of the secondary and virgin curves remains quite small. However, we should emphasize that abnormal hysteretic behavior of wings can be explained if we assume that the influence of the proximity effects, and hence the shift magnitude, increases at a larger field modulus.

The crucial problem is the origin of ferromagnetic ordering in our nanocomposite on the nanometer scale. Bulk indium and AgIn_2_ intermetallic are not magnetic. However, it is known that some metals, which are not ferromagnetic in bulk, show ferromagnetism at decreasing size. Magnetization isotherms typical for ferromagnetics were reported for small particles of Au, Pt, Pd, and Sn [47,48,49,50]. The emergence of ferromagnetism was related to the appearance of uncompensated spins at the interface. For small metallic particles, the *M*(*H*) hysteresis loops demonstrated rather weak remanence and saturation magnetization. The magnetization isotherm shown in Figure 4d has similar features. The inset to Figure 4d allows us to estimate the saturation magnetization and to evaluate the magnetic moment per the nominal formula unit of the alloy. It was found to be equal to 3.6 × 10^−4^ $\mu_{B}$, where $\mu_{B}$ is the Bohr magneton. This value is close to the relevant value found for small Pt particles in [49]. We can suggest that the interfaces between the indium and AgIn_2_ segregates serve as sources of uncompensated spins in the nanocomposite consisting of porous glass and In–Ag alloy.

Figure 2 shows noticeable thermal hysteresis between the FCC and FCW curves below the second superconducting transition. Similar hysteresis was observed occasionally in some dirty type-II superconductors, for instance, for polycrystalline and powder Nb_3_Sn and YBa_2_Cu_3_O_7_ samples [51] and an opal template filled with the Ga-Sn alloy [12]. The relevant theoretical model was considered in [52,53]. The assumption of the magnetic flux freezing under cooling was made. According to the shape of the *M*(*H*) isotherms shown in Figure 4 and Figure 6, the most amount of indium segregates are in the intermediate state just below the second transition, even at quite low magnetic fields. While no theoretical models are known for the hysteresis between the FCC and FCW curves in the intermediate state, we can suggest that the hysteresis is also related to the freezing of the magnetic flux at cooling the nanocomposite.

## 5. Conclusions

The magnetization studies suggest that two superconducting transitions in the nanostructured In–Ag alloy confined to a porous glass template correspond to formation within pores of strongly linked indium segregates and a small amount of amorphous indium or fine, weakly linked indium particles. The AgIn_2_ segregates are nonsuperconducting. The *M*(*H*) isotherms measured above the superconducting transitions and the bifurcation of the ZFC and FCC curves evidenced the emergence of ferromagnetism in the porous glass/In–Ag nanocomposite, which was most likely induced at the interface between the In and AgIn_2_ segregates. The coexistence of ferromagnetism and superconductivity at the nanometer scale provoked shifts in the magnetization branches with respect to each other. The shift sign showed the dominant role of the stray fields.

## Data Availability

Data are contained within the article or Supplementary Materials.

## Supplementary Materials

The following supporting information can be downloaded at: https://www.mdpi.com/article/10.3390/nano14221792/s1, Figure S1: The central part of the M(H) isotherm at 1.8 K for the bulk In-Ag alloy before the corrections for the residual fields of the superconducting magnet (black symbols and lines) and after corrections (red symbols and lines); Figure S2: Main part of the temperature dependences of magnetization obtained in the porous glass/In-Ag alloy nanocomposite obtained under the ZFC and FCC protocols.

## References

[1] Wördenweber R., Moshchalkov V., Bending S., Tafuri F. (2017). Superconductors at the Nanoscale: From Basic Research to Applications.

[2] Xiong Y., Lu X. (2015). Metallic Nanostructures: From Controlled Synthesis to Applications.

[3] Sun L.G., Wu G., Wang Q., Lu J. (2020). Nanostructural Metallic Materials: Structures and Mechanical Properties. Mater. Today.

[4] Ochirkhuyag N., Matsuda R., Song Z., Nakamura F., Endo T., Ota H. (2021). Liquid Metal-Based Nanocomposite Materials: Fabrication Technology and Applications. Nanoscale.

[5] Li X., Lu L., Li J., Zhang X., Gao H. (2020). Mechanical Properties and Deformation Mechanisms of Gradient Nanostructured Metals and Alloys. Nat. Rev. Mater..

[6] Nefedov D.Y., Podorozhkin D.Y., Charnaya E.V., Uskov A.V., Haase J., Kumzerov Y.A., Fokin A.V. (2019). Liquid–Liquid Transition in Supercooled Gallium Alloys under Nanoconfinement. J. Phys. Condens. Matter.

[7] Gafner Y.Y., Gafner S.L., Zamulin I.S., Redel L.V., Samsonov V.M. (2013). Possible Mechanisms of Increase in Heat Capacity of Nanostructured Metals. Phys. Solid State.

[8] Murashkin M.Y., Sabirov I., Sauvage X., Valiev R.Z. (2016). Nanostructured Al and Cu Alloys with Superior Strength and Electrical Conductivity. J. Mater. Sci..

[9] Wu P.C., Kim T., Suvorova A., Giangregorio M., Saunders M., Bruno G., Brown A.S., Losurdo M. (2011). GaMg Alloy Nanoparticles for Broadly Tunable Plasmonics. Small.

[10] Bose S. (2023). A Review of Superconductivity in Nanostructures—From Nanogranular Films to Anti-Dot Arrays. Supercond. Sci. Technol..

[11] Lang W. (2024). Nanostructured Superconductors. Encyclopedia of Condensed Matter Physics.

[12] Likholetova M.V., Charnaya E.V., Shevchenko E.V., Lee M.K., Chang L.-J., Kumzerov Y.A., Fokin A.V. (2023). Magnetic Studies of Superconductivity in the Ga-Sn Alloy Regular Nanostructures. Nanomaterials.

[13] Gokhfeld D.M., Koblischka M.R., Koblischka-Veneva A. (2020). Highly Porous Superconductors: Synthesis, Research, and Prospects. Phys. Met. Metallogr..

[14] Lee M.K., Charnaya E.V., Mühlbauer S., Jeng U., Chang L.J., Kumzerov Y.A. (2021). The Morphologic Correlation between Vortex Transformation and Upper Critical Field Line in Opal-Based Nanocomposites. Sci. Rep..

[15] Ciou Y.S., Lee M.K., Charnaya E.V., Tien C., Chang L.J., Kumzerov Y.A., Samoylovich M.I. (2013). Superconductivity in Sn Nanocomposites. Supercond. Sci. Technol..

[16] Watson J.H.P. (1966). Critical Magnetic Field and Transition Temperature of Synthetic High-Field Superconductors. Phys. Rev..

[17] Gokhfeld D.M., Popkov S.I., Bykov A.A. (2019). Analog of the Intertype Superconductivity in Nanostructured Materials. Phys. C.

[18] Likholetova M.V., Charnaya E.V., Kumzerov Y.A., Fokin A.V., Grigorieva N.R., Mikushev V.M., Shevchenko E.V. (2023). Coexistence of Superconductivity and Ferromagnetism in a Nanocomposite Based on Porous Glass with Nickel and Indium Inclusions. Phys. Solid State.

[19] Ekin J.W. (2022). Superconductor to Normal-Metal Contacts. Handbook of Superconductivity.

[20] Made R.I., Gan C.L., Yan L.L., Yu A., Yoon S.W., Lau J.H., Lee C. (2009). Study of Low-Temperature Thermocompression Bonding in Ag-In Solder for Packaging Applications. J. Electron. Mater..

[21] Rossi P.J., Zotov N., Mittemeijer E.J. (2016). Kinetics of Intermetallic Compound Formation in Thermally Evaporated Ag-In Bilayers. J. Appl. Phys..

[22] Granqvist C.G. (1975). Superconductivity of Amorphous Indium with Silver Impurities. Solid State Commun..

[23] Granqvist C.G., Claeson T. (1979). Superconducting Transition Temperatures of Vapour Quenched Ag-In and Ag-Sn Multilayers. Solid State Commun..

[24] Okamoto H., Massalski T.B., Okamoto H., Schlesinger M.E., Mueller E.M. (1990). Binary Alloy Phase Diagrams.

[25] Moser Z., Gasior W., Pstrus J., Zakulski W., Ohnuma I., Liu X.J., Inohana Y., Ishida K. (2001). Studies of the Ag-In Phase Diagram and Surface Tension Measurements. J. Electron. Mater..

[26] Kroupa A., Dinsdale A.T., Watson A., Vrestal J., Vízdal J., Zemanova A. (2007). The Development of the COST 531 Lead-Free Solders Thermodynamic Database. JOM.

[27] Zhang J.-F., Guo P.-J., Gao M., Liu K., Lu Z.-Y. (2019). β—RhPb 2: A Topological Superconductor Candidate. Phys. Rev. B.

[28] Lazarev B.G., Semenenko E.E., Sudovtsov A.I., Kuz’menko V.M. (1966). Maximum Critical Magnetic Fields in Superconducting Metals. Soviet Phys. Doklady.

[29] Tien C., Wur C.S., Lin K.J., Charnaya E.V., Kumzerov Y.A. (2000). Double-Step Resistive Superconducting Transitions of Indium and Gallium in Porous Glass. Phys. Rev. B.

[30] Graf M.J., Huber T.E., Huber C.A. (1992). Superconducting Properties of Indium in the Restricted Geometry of Porous Vycor Glass. Phys. Rev. B.

[31] Shamshur D.V. (2005). Electrical Conductivity and Superconductivity of Ordered Indium–Opal Nanocomposites. Phys. Solid State.

[32] Li W.-H., Yang C.C., Tsao F.C., Wu S.Y., Huang P.J., Chung M.K., Yao Y.D. (2005). Enhancement of Superconductivity by the Small Size Effect in In Nanoparticles. Phys. Rev. B.

[33] Shaw R.W., Mapother D.E., Hopkins D.C. (1960). Critical Fields of Superconducting Tin, Indium, and Tantalum. Phys. Rev..

[34] Provost J., Paumier E., Fortini A. (1974). Shape Effects on the Magnetization of Superconducting Lead at 4.2K. J. Phys. F Met. Phys..

[35] Satariano R., Parlato L., Vettoliere A., Caruso R., Ahmad H.G., Miano A., Palma L.D., Salvoni D., Montemurro D., Granata C. (2021). Inverse Magnetic Hysteresis of the Josephson Supercurrent: Study of the Magnetic Properties of Thin Niobium/Permalloy (Fe_20_Ni_80_) Interfaces. Phys. Rev. B.

[36] Kapran O.M., Golod T., Iovan A., Sidorenko A.S., Golubov A.A., Krasnov V.M. (2021). Crossover between Short- and Long-Range Proximity Effects in Superconductor/Ferromagnet/Superconductor Junctions with Ni-Based Ferromagnets. Phys. Rev. B.

[37] Parlato L., Caruso R., Vettoliere A., Satariano R., Ahmad H.G., Miano A., Montemurro D., Salvoni D., Ausanio G., Tafuri F. (2020). Characterization of Scalable Josephson Memory Element Containing a Strong Ferromagnet. J. Appl. Phys..

[38] Buzdin A.I. (2005). Proximity Effects in Superconductor-Ferromagnet Heterostructures. Rev. Mod. Phys..

[39] Fermin R., Van Dinter D., Hubert M., Woltjes B., Silaev M., Aarts J., Lahabi K. (2022). Superconducting Triplet Rim Currents in a Spin-Textured Ferromagnetic Disk. Nano Lett..

[40] Aladyshkin A.Y., Silhanek A.V., Gillijns W., Moshchalkov V.V. (2009). Nucleation of Superconductivity and Vortex Matter in Superconductor—Ferromagnet Hybrids. Supercond. Sci. Technol..

[41] Krivoruchko V.N., Koshina E.A. (2002). Inhomogeneous Magnetism Induced in a Superconductor at a Superconductor-Ferromagnet Interface. Phys. Rev. B.

[42] Bergeret F.S., Volkov A.F., Efetov K.B. (2004). Induced Ferromagnetism Due to Superconductivity in Superconductor-Ferromagnet Structures. Phys. Rev. B.

[43] Bergeret F.S., Yeyati A.L., Martín-Rodero A. (2005). Inverse Proximity Effect in Superconductor-Ferromagnet Structures: From the Ballistic to the Diffusive Limit. Phys. Rev. B.

[44] Löfwander T., Champel T., Durst J., Eschrig M. (2005). Interplay of Magnetic and Superconducting Proximity Effects in Ferromagnet-Superconductor-Ferromagnet Trilayers. Phys. Rev. Lett..

[45] Mironov S., Mel’nikov A.S., Buzdin A. (2018). Electromagnetic Proximity Effect in Planar Superconductor-Ferromagnet Structures. Appl. Phys. Lett..

[46] Devizorova Z., Mironov S.V., Mel’nikov A.S., Buzdin A. (2019). Electromagnetic Proximity Effect Controlled by Spin-Triplet Correlations in Superconducting Spin-Valve Structures. Phys. Rev. B.

[47] Crespo P., Litrán R., Rojas T.C., Multigner M., De La Fuente J.M., Sánchez-López J.C., García M.A., Hernando A., Penadés S., Fernández A. (2004). Permanent Magnetism, Magnetic Anisotropy, and Hysteresis of Thiol-Capped Gold Nanoparticles. Phys. Rev. Lett..

[48] Yamamoto Y., Miura T., Suzuki M., Kawamura N., Miyagawa H., Nakamura T., Kobayashi K., Teranishi T., Hori H. (2004). Direct Observation of Ferromagnetic Spin Polarization in Gold Nanoparticles. Phys. Rev. Lett..

[49] Sakamoto Y., Oba Y., Maki H., Suda M., Einaga Y., Sato T., Mizumaki M., Kawamura N., Suzuki M. (2011). Ferromagnetism of Pt Nanoparticles Induced by Surface Chemisorption. Phys. Rev. B.

[50] Li W.-H., Wang C.-W., Li C.-Y., Hsu C.K., Yang C.C., Wu C.-M. (2008). Coexistence of Ferromagnetism and Superconductivity in Sn Nanoparticles. Phys. Rev. B.

[51] Hyun O.B. (1993). Experimental Aspects of Flux Expulsion in Type-II Superconductors. Phys. Rev. B.

[52] Krusin-Elbaum L., Malozemoff A.P., Cronemeyer D.C., Holtzberg F., Clem J.R., Hao Z. (1990). New Mechanisms for Irreversibility in High-*T_c_* Superconductors (Invited). J. Appl. Phys..

[53] Clem J.R., Hao Z. (1993). Theory for the Hysteretic Properties of the Low-Field Dc Magnetization in Type-II Superconductors. Phys. Rev. B.

