# Supplementary material for: Coexistence of Superconductivity and Magnetic Ordering in the In–Ag Alloy Under Nanoconfinement
Source: Nanomaterials (Basel). 2024 Nov 7;14(22):1792. doi: 10.3390/nano14221792 (PMC11597133; doi:10.3390/nano14221792)
Supplement: Supplementary file 1 [file nanomaterials-14-01792-s001.zip › nanomaterials-3283909-supplementary.pdf]

Coexistence of superconductivity and magnetic ordering in the In-Ag alloy under nanoconfinement

Marina V. Likholetova [1], Elena V. Charnaya [1,*], Evgenii V. Shevchenko [1], Yurii A. Kumzerov [2] and Aleksandr V. Fokin [2]

[1] Physics Department, St. Petersburg State University, 198504 St. Petersburg, Russia; m.likholetova@spbu.ru (M.V.L.); e.shevchenko@spbu.ru (E.V.S.)
[2] Ioffe Institute, 194021 St. Petersburg, Russia; yu.kumzerov@mail.ioffe.ru (Y.A.K.); midbarzin@yandex.ru (A.V.F.)

Supplementary materials

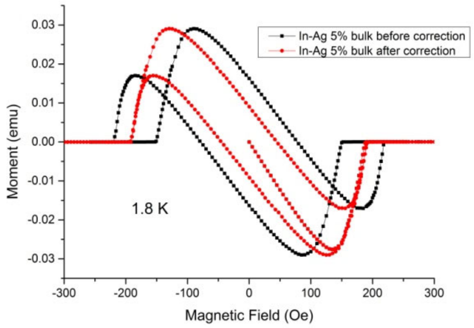

Figure S1. The central part of the M(H) isotherm at 1.8 K for the bulk In-Ag alloy before the corrections for the residual fields of the superconducting magnet (black symbols and lines) and after corrections (red symbols and lines).

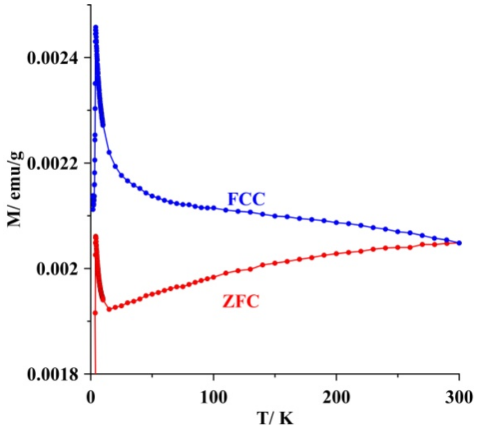

Figure S2. Main part of the temperature dependences of magnetization obtained in the porous glass/In-Ag alloy nanocomposite obtained under the ZFC and FCC protocols.
